# Recalcitrant chronic bladder pain and recurrent cystitis but negative urinalysis: What should we do?

**DOI:** 10.1007/s00192-018-3569-7

**Published:** 2018-03-20

**Authors:** Sheela Swamy, William Barcella, Maria De Iorio, Kiren Gill, Rajvinder Khasriya, Anthony S. Kupelian, Jennifer L. Rohn, James Malone-Lee

**Affiliations:** 1 Centre for Nephrology, Division of Medicine, UCL, London, UK; 2 Department of Statistical Science, Faculty of Mathematics and Science, UCL, London, UK; 3 Hillingdon Hospitals NHS Trust, London, UK; 4 University College London Hospitals NHS Trust, London, UK

**Keywords:** Chronic bladder pain, Bladder pain syndrome, Recalcitrant LUTS, Chronic UTI, Recurrent UTI, Interstitial cystitis

## Abstract

**Purpose:**

Lower urinary tract symptoms (LUTS) may be associated with chronic urinary tract infection (UTI) undetected by routine diagnostic tests. Antimicrobial therapy might confer benefit for these patients.

**Materials and methods:**

Over 10 years, we treated patients with chronic LUTS. Pyuria was adopted as the principal biomarker of infection. Urinary leucocyte counts were recorded from microscopy of fresh midstream urine (MSU) samples. Antibiotics were prescribed and the prescription adjusted to achieve a measurable clinical response and a reduction in pyuria.

**Results:**

We treated 624 women [mean age = 53.4 years; standard deviation (SD) = 18] with chronic LUTS and pyuria. Mean duration of symptoms prior to presentation was 6.5 years. Only 16% of MSU cultures submitted were positive (≥10^5^ cfu ml^-1^). Mean treatment length was 383 days [SD = 347; 95% confidence interval (CI) = 337–428]. Treatment was associated with a reduction in total LUTS (*F* = 98; *p* = 0.0001), 24-h frequency (*F* = 75; *p* = 0.0001), urinary urgency (*F* = 90; *p* = 0.0001), lower urinary tract pain (*F* = 108;* p* = 0.0001), voiding symptoms (*F* = 10; *p* = 0.002), and pyuria (*F* = 15.4;* p* = 0.0001). Full-dose first-generation antibiotics for UTI, such as cefalexin, nitrofurantoin, or trimethoprim, were combined with methenamine hippurate. We recorded 475 adverse events (AEs) during 273,762 treatment days. There was only one serious adverse event (SAE). We observed no increase in the proportion of resistant bacterial isolates.

**Conclusion:**

This large case series demonstrates that patients with chronic LUTS and pyuria experience symptom regression and a reduction in urinary tract inflammation associated with antimicrobial therapy. Disease regression was achieved with a low frequency of AEs. These results provide preliminary data to inform a future randomized controlled trial (RCT).

## Introduction

There is growing awareness that urinary tract infection (UTI), undetected by routine diagnostics, might play a causal role in the generation of lower urinary tract symptoms (LUTS) [1, 2]. This proposal is controversial because the exclusion of UTI is a key step in every guideline concerned with the management of LUTS [3]. As a result, LUTS have become synonymous with noninfective disease. However, there is increasing evidence that patients presenting with LUTS may harbour a UTI despite negative tests. The problem lies with the diagnostic criteria used in culture-based diagnosis. A significant body of published literature points to the inherent flaws of quantitative urinary microbiological analysis, in which thresholds between 10^3^ cfu ml^−1^ and 10^6^ cfu ml^−1^ of a pure growth of a single urinary pathogen are employed to diagnose UTI [4, 5]. Dipstick urinalysis performs equally poorly, hampered by insensitivity and spectrum bias [6, 7]. These tests cannot reliably exclude UTI and do not take into account differences in bacterial strain virulence, host genetic variability, intracellular bacterial reservoirs, or even urine dilution due to high fluid intake before the test.

Pyuria, detected by microscopy of a fresh midstream urine (MSU) specimen, is the most sensitive surrogate marker of UTI [6]. It circumvents the problems associated with quantitative bacterial culture, and its value in the diagnosis of UTI is recognised by international practice guidelines [8]. Whilst ≥10 wbc μl^−1^ is employed almost universally to diagnose UTI, contemporary data cast doubt on this threshold in patients with LUTS [6, 9]. In the symptomatic patient, controlled studies have demonstrated that lower pyuria counts of 1–9 wbc μl^−1^ are associated with an increase in independent inflammatory and microbiological markers of UTI [6, 10]. Thus, lower levels of pyuria may also indicate infection and immune activation.

The diagnostic picture has become even more complex with the recent discovery that UTI can legitimately involve polymicrobial infection; mixed growth cultures do not necessarily reflect contamination [5, 11]. What’s more, advances in enhanced culture and genomics technology have revealed that even the normal bladder is not sterile and that a bona fide, possibly protective, urinary microbiota can be described [5, 11]. Uncertain diagnosis is not the only area that complicates UTI management. The treatment of acute cystitis also has its limitations. A Cochrane review reported symptomatic and microbiological failure rates of 37 and 28% 4–10 weeks after treatment [12]. Amongst healthy young women with their first UTI, the risk of recurrence within 6 months is 24%. If they have a history of one or more UTIs, the risk of recurrence rises to 70% in that same year [13]. In a Canadian surveillance study, 14% of 30,851 residents with UTI experienced more than one episode during the 2-year study period, and 2% had six or more episodes [14].

Taken together, these findings are of real concern, and whilst guidance on the diagnosis and management of UTI is increasingly prescriptive, one size does not necessarily fit all. We hypothesise that chronic LUTS may result from urinary infection falling below the routine culture threshold and that antibiotic treatment could confer benefit. Indeed, Stamm et al. published an RCT reporting success from a 10-day regimen of doxycycline for culture-negative patients with acute frequency and dysuria who had microscopic pyuria (using ≥8 wbc ul^−1^ as their threshold) [15]. Further studies are needed to support these findings.

In 2004, we started to use antibiotics to treat patients with chronic LUTS who demonstrated microscopic pyuria, despite negative dipstick testing and negative MSU cultures employing a 10^5^ cfu ml^−1^ threshold. Using sensitive microbiological methods, we have since generated data demonstrating widespread bacterial infection in these patients [1]. Other groups have also reported similar findings [4]. Our clinical approach sought to manage patients towards symptom resolution and clearance of pyuria by combining antibiotic treatment with careful outpatient monitoring. This paper describes the evolution of our clinical practice as a result of these observations.

## Methods

These data represent a large case series of women treated in a single tertiary centre from 2004 to 2014. All women with chronic LUTS and pyuria ≥1 wbc μl^−1^ who agreed to antibiotic therapy provided symptom and urinalysis data. This group included patients with existing diagnoses of overactive bladder (OAB), bladder pain syndrome/interstitial cystitis (BPS/IC), voiding symptoms, and recurrent UTI (rUTI). Stress urinary incontinence (SUI) was a common nondominant symptom but was not a therapeutic target for antibiotic treatment. All patients presenting underwent a standardised clinical assessment. Demographic data, medical comorbidities and concurrent medication were recorded, together with details of previous treatments, in our electronic patient database. LUTS was characterised using a 39-question inventory developed and validated at this centre [16] (Table 1); urinary urgency and lower urinary tract pain subscales were validated as independent measures [17, 18]. Microscopic pyuria was enumerated from a freshly collected clean-catch MSU specimen, which was submitted for routine culture employing a ≥ 10^5^ cfu ml^−1^ diagnostic threshold. Antibiotic sensitivity testing was conducted on all bacterial isolates.

**Table 1:** Validated lower urinary tract sysptom (LUTS) questionnaire

**Urinary urgency**
1. Urgency
2. Urgency incontinence
3. Latchkey urgency
4. Latchkey urgency incontinence
5. Waking urgency
6. Waking urgency incontinence
7. Running water urgency
8. Running water urgency incontinence
9. Cold urgency
10. Anxiety urgency
11. Premenstrual aggravation
**Stress urinary incontinence**
12. Cough/sneeze incontinence
13. Exercise incontinence
14. Laughing incontinence
15. Passive Incontinence
16. Bending incontinence
17. Standing incontinence
18. Lifting incontinence
19. Pre-cough preparation
**Voiding symptoms**
20. Hesitancy
21. Reduced stream
22. Intermittent stream
23. Straining to void
24. Terminal dribbling
25. Postmicturition dribbling
26. Double voiding
**Pain symptoms**
27. Suprapubic pain
28. Filling bladder pain
29. Voiding bladder pain
30. Postvoid bladder pain
31. Pain relieved by voiding
32. Pain partially relieved by voiding
33. Pain unchanged by voiding
34. Loin pain
35. Iliac fossa pain
36. Pain radiating to genitals
37. Pain radiating to legs
38. Dysuria
39. Urethral pain

Antibiotic therapy was not contingent on positive dipstick urinalysis or routine MSU cultures. As previously described, these tests are insufficiently sensitive to exclude UTI and were not used to arbitrate treatment decisions. Results of MSU culture were used to monitor bacterial resistance rates only. Written information was provided explaining the justification for treatment, expected clinical outcomes and potential adverse effects. Patients understood that they were being treated outside of standard guidelines. The clinical lead for the unit took sole responsibility for all off-label prescribing.

The assessments conducted at presentation were repeated at every follow-up visit. Duration of follow-up was variable, as treatment response dictated the number of review visits. All data, up to the point of patient discharge, was included in the analysis. The Patient Global Impression of Improvement (PGI-I) scale was used to measure treatment response [19, 20]. Adverse events (AEs) were recorded at every patient interaction and graded using the Common Terminology Criteria for Adverse Events (CTC-AE), version 4.3. Clinical management evolved during the study period. Treatment was initiated with first-line antibiotics used for UTI, including nitrofurantoin, trimethoprim and cefalexin. Patients were reviewed for response at 14 days, or earlier if AEs were encountered. Treatment modifications were tailored to efficacy and tolerance [21]. The urinary antiseptic agent methenamine hippurate was used as an adjunct, and some patients required combination antibiotic therapy. Wherever possible, we tried to forge our observations into useful amendments of these personalised regimens.

An effective, tolerated treatment was continued until symptoms were ameliorated and the inflammatory response in urine had resolved. Treatment was then discontinued. If the symptoms recurred, treatment was reinstated. This cycle was repeated until there was no symptomatic deterioration after antibiotic withdrawal for 12 weeks. At this point, patients were asked to initiate short-course, self-start antibiotic treatment at the first sign of any recurrence of symptoms which was identical to the self-start regimens recommended for rUTI. Symptom-based antimicrobial treatment, introduced early in the infection cycle, was advocated to prevent chronic symptom recurrence [22].

### Statistical analysis

All statistical analyses were conducted using IBM SPSS 22 (IBM, NY, New York, USA). Changes in outcome variables were analysed using a mixed-models, linear regression analysis within a repeated measures design. The time interval from first attendance at each clinic visit varied between patients, and intervals could vary for individual patients. To address this, the effect of the number of days from first attendance was controlled in all models involving multiple attendances. Time from first visit was entered as a covariate, accounting for patient-specific random effects. Data were indexed by patient and visit number using a main effects model and random intercept. Ordinal regression was used to analyse PGI-I responses recorded on the last visit only. Comparison of nonparametric data used the Kruskall–Wallis or Mann–Whitney* U* tests.

### Ethical approval

Validated symptom and biomarker data were collected in accordance with a protocol approved by the East Central London Regional Ethics Committee (REC1) (Ref: 11/H0721/7).

## Results

### Patients

A total of 1996 women presented to the clinical service between 2004 and 2014: 433 attended just once for urodynamic studies or urinalysis, and these patients were neither treated in the centre nor followed up. A further 444 women were treated for OAB and did not demonstrate pyuria or presented with SUI as their only symptom. These women were not treated with antibiotics. After these exclusions, 624 women [mean age = 53.4 years; standard deviation (SD) = 18] who demonstrated pyuria ≥1 wbc μl^−1^ at presentation were included in the analysis.

Patients described longstanding LUTS prior to their referral to this service (mean duration = 6.5 years; SD = 6.3). Many women had an established diagnosis of OAB or BPS/IC from elsewhere. Urinary urgency symptoms were described by 73% of women, whilst voiding symptoms and lower urinary tract pain affected 71% and 65%, respectively; 43% of women described SUI. Patient demographics and symptoms are summarised in Table 2.

**Table 2:** Patient demographics and summarised symptom data collected at first attendance

Demographics	Median	Mean	SD	SEM	95% CI
Age (years)	54.0	53.4	18	0.7	52–54.8
Symptom duration (years)	4.0	6.4	6.3	0.3	5.9–7.0
24-h urinary frequency	10	10.8	5.2	0.2	10.4–11.2
24-h incontinence episodes	0.4	1.2	2.0	0.1	1.1–1.4
Total LUTS symptom score (0–39)	11.0	11.1	6.3	0.3	10.6–11.6
Urgency subscale score (0–11)	4.0	3.8	3.2	0.1	3.5–4.0
Pain subscale score (0–13)	3.0	3.2	3.2	0.1	3.0–3.5
Voiding subscale score (0–7)	3.0	2.9	2.4	0.1	2.7–3.1
SUI subscale score (0–8)	0.0	1.2	1.9	0.1	1.0–1.3

*LUTS* lower urinary tract symptoms,* SUI* stress urinary incontinence, * SD* standard deviation,* SEM* standard error of mean,* CI* confidence interval

### Dipstick and urine culture

We performed 1988 dipstick analyses: 558 (28%) demonstrated trace or greater leucocyte esterase; 138 (7%) were nitrite positive. However, 1433 (72%) of these samples showed pyuria on direct microscopy. Of the 2209 MSU cultures performed during observation, only 362 (16%) were positive using the threshold of ≥10^5^ cfu ml^−1^, although microscopic pyuria was recorded in 1741 (79%) of these samples.

### Antibiotic use

Our prescribing practice evolved over the course of the observation period as we scrutinised our treatment–response data. This led to treatment regimens being simplified and refined as data were collected. In 2014, when data collection ceased, 80% of patients were being treated with 12 antibacterial regimens. Six of these consisted of methenamine hippurate combined with one antibiotic, most commonly a first-generation antimicrobial such as cefalexin, nitrofurantoin or trimethoprim. Full-dose treatment was administered. We identified a cluster of patients with marked urethral pain and low-level pyuria whose symptoms preferentially responded to macrolide or tetracycline, perhaps suggestive of a fastidious microorganism.

### Treatment duration and efficacy

We tested the need for ongoing treatment empirically by stopping antimicrobial therapy. Treatment cessation was permitted once any reduction in LUTS had reached a steady state and pyuria had cleared. If symptoms recurred, the occurrence was documented and treatment reinstated. Thus, we stopped treatment 858 times and restarted 633 (74%) times on recurrence. Amongst patients with pain symptoms, relapses were associated with significantly higher pain scores (mean = 4.2; 95% CI = 3.6–4.9) compared with their symptoms at the beginning of treatment (mean = 2.7; 95% CI = 2.2–3.2) (*p* = 0.001).

Two hundred and twenty-five women completed treatment and were discharged. The median number of patient visits was five (mean = 6.6; SD = 5), with 40% of women discharged after four visits and 80% within ten. Mean treatment length was 383 days, with a significant variation in duration (SD = 347; 95% CI = 337–428). Some patients required long-term therapy, as attempts to withdraw treatment were associated with relapse. Others were treated successfully but requested long-term monitoring due to anxieties about disease recurrence.

**Fig. 1:**
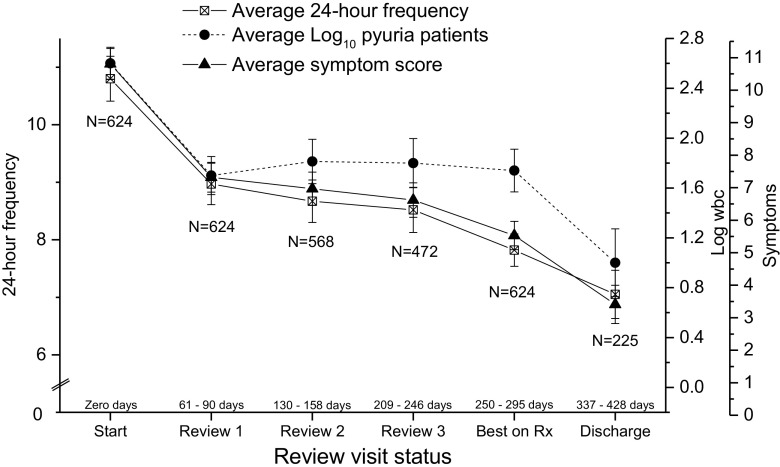
Change in urinary frequency, log_10_ pyuria and total lower urinary tract symptoms (LUTS) plotted against visit status: mean and 95% confidence interval (CI)

Figure 1 shows a plot of 24-h urinary frequency, total LUTS and pyuria at time points (A) baseline, (B) three subsequent review visits, (C) optimum state on treatment and (D) discharge. There was a significant reduction in 24-h urinary frequency (*F* = 75; *p* = 0.0001), total LUTS (*F* = 98;* p* = 0.0001) and log_10_ pyuria (*F* = 15.4;* p* = 0.0001). Incontinence episodes did not change significantly (*F* = 1.8; *p* = .18). Optimum treatment-associated effects amongst patients who were not discharged are represented by data at time point (C).

**Fig. 2:**
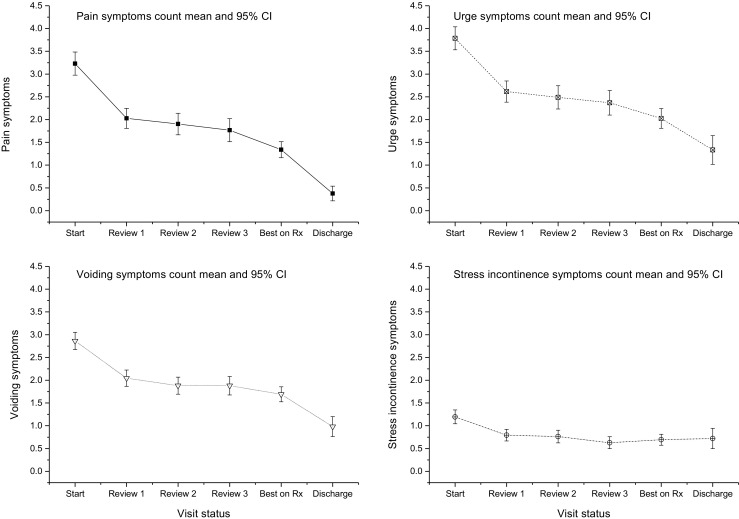
Change in individual lower urinary tract symptom (LUTS) subscales plotted against visit status.

Separate analyses demonstrating the change in individual symptom subscales associated with treatment are presented in Fig. 2. Antibiotic treatment was associated with a significant reduction in urinary urgency (*F* =  90; *p* = 0.0001), lower urinary tract pain (*F* = 108;* p* = 0.0001) and voiding symptoms (*F* = 10; *p* = 0.002). Symptoms of SUI were unchanged during treatment (*F* = 1.4; *p* = 0.24) (Table 3).

**Table 3:** Changes in lower urinary tract symptoms (LUTS) associated with treatment using a mixed-models linear regression analysis within a repeated measures design

Dependent variable: outcome measure	*F* statistic	Fisher’s* p*
Log_10_ wbc μl^-1^	15.4	0.0001
24-h urinary frequency	75.0	0.0001
24-h incontinence episodes	1.8	.18
Total LUTS symptom score	98.0	0.0001
Urgency subscale score	90.0	0.0001
Pain subscale score	108.6	0.0001
Voiding subscale score	10.1	0.002
SUI subscale score	1.4	.24

*Independent variable* time from first visit in days, *Repeated measures* visit number

The PGI-I responses demonstrated a significant improvement over the treatment period (χ^2^ = 2272;* df* = 5; *p* = 0.001); 84% of women rated their condition as much better (20%) or very much better (64%). These data demonstrate that long-term antibiotic treatment is associated with resolution of chronic LUTS and pyuria, which is an independent surrogate marker of infection. These findings were accompanied by PGI-I responses indicating that most patients perceived these symptomatic improvements to be clinically meaningful.

**Fig. 3:**
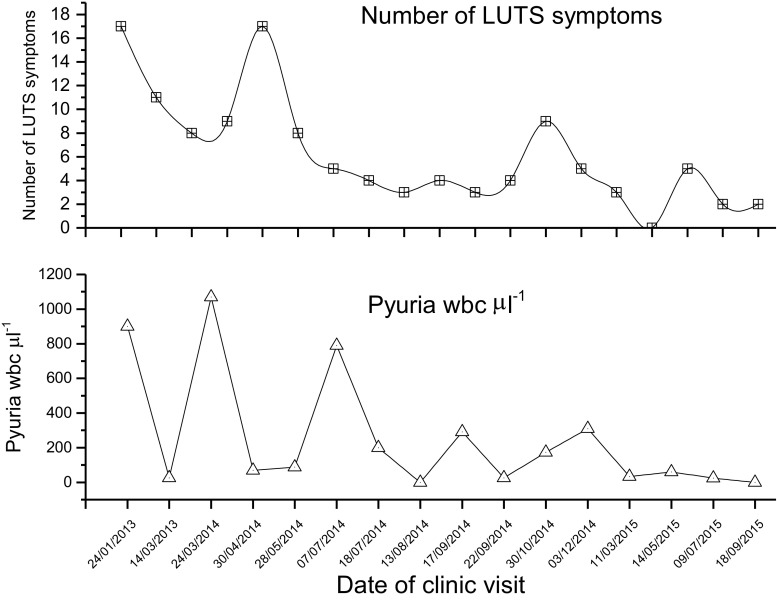
Symptoms and pyuria of one illustrative patient showing the associated damped oscillation that occurred in 73% of women during treatment

Plots of pyuria and symptoms in individual patients for each visit showed a typical response pattern in 73% of cases, termed a damped oscillation (Fig. 3). This features a series of oscillations of decreasing amplitude; 10% of patients demonstrated the converse, and this was associated with symptomatic deterioration. Ten per cent showed a rapid, uninterrupted fall to baseline (critically damped oscillation) and 7% were unclassifiable.

Baseline variables of age, duration of symptoms, urinary frequency/incontinence and pyuria did not predict treatment outcome. Higher pain scores at presentation were predictive of greater maximal symptom palliation associated with antibiotic therapy (b = 0.57, *p* = 0.029). Higher scores for urinary urgency (b = 1.1, *p* = 0.001) and voiding symptoms (b = 1.0, *p* = 0.005) predicted less favourable responses, although the magnitude of these effects was small.

### Adverse events

Two hundred and sixty-six patients reported 475 AEs during 273,762 treatment days. Except one, all AEs were classified as mild or moderate (CTC AE grades 1–2). A change in antibiotic was instituted in all cases. AEs are listed by system in Table 4 with the CTC AE grading definitions.

**Table 4:** Adverse events

Type	Frequency (%)
Total	475 (100)
General reactions	255 (53.7)
Malaise or nonspecific systemic upset	195 (41.1)
Cutaneous reactions	47 (9.9)
Pruritus	13 (2.7)
Gastrointestinal disorders	84 (17.6)
Diarrhoea*	42 (8.8)
Nausea	33 (6.9)
Constipation	5 (1.1)
Vomiting	4 (0.8)
Respiratory disorders	50 (10.7)
Dyspnoea	38 (8.2)
Cough	11 (2.3)
Eosinophilic pneumonitis and fibrosis**	1 (0.2)
Musculoskeletal disorders	39 (8.2)
Arthralgia	39 (8.2)
Central nervous system disorders	19 (4.0)
Headache	17 (3.6)
Paraesthesia	2 (0.4)
Laboratory abnormalities	28 (5.9)
Transaminitis	28 (5.9)

Common Terminology Criteria for Adverse Events (CTC-AE) grading system: (1) mild—asymptomatic or mild symptoms, intervention not required; (2) moderate—minimal intervention indicated; (3) severe or medically significant but not immediately life threatening, hospitalisation or prolongation of hospitalisation indicated; (4) life threatening—urgent intervention indicated; (5) death

*Six cases of* Clostridium-difficile*-toxin-positive diarrhoea; 1 case of* C.-difficile*-toxin-positive antigen

**Serious adverse event

To our knowledge, the only SAE (CTC-AE grade 3) was an eosinophilic pneumonitis associated with the use of nitrofurantoin. A patient with severe LUTS, only ameliorated by nitrofurantoin, was exposed intermittently to the drug over 8 years, with numerous attempts at cessation and use of alternatives. She developed a sudden eosinophilic pneumonitis with some fibrosis. Six cases of *Clostridium-difficile*-toxin-positive diarrhoea and one case of diarrhoea expressing *C.difficile* antigen was seen during treatment. All were treated as outpatients. Seven patients with a history of *C. difficile* diarrhoea were managed without recurrence. No other AEs were recorded.

### Antibiotic resistance

We analysed data from all 362 positive MSU cultures. The median number of antibiotics to which the isolate was resistant remained at one over all visits [interquartile range (IQR) 0–2 for visits one and two, and 0–3 for the third and subsequent visits). These differences were not significant (Kruskal–Wallis χ^2^ = 2.5;* df* = 3; *p* = 0.47). These data demonstrate that long-term antibiotic use, as practiced using our methods, does not appear to generate antibiotic resistance amongst uropathogens detected by standard urine culture.

## Discussion

Our clinical experience over a decade with a large cohort of patients demonstrates the promise that antibiotic therapy may hold for treating symptoms previously ascribed to a noninfectious aetiology. In the process, it strengthens the hypothesis that UTI might be implicated in the generation of chronic LUTS. In 2008, it was estimated that nearly 45% of the world’s population were affected by LUTS, and this is expected to increase as our population ages. Urinary urgency incontinence affects 15–22% of people in the USA [23], with a staggering estimated cost of US$76 billion annually. If even a fraction of these patients have a treatable bacterial aetiology, their care might be transformed.

The principal limitation of this work is that it represents an evolution of clinical practice rather than an intervention study. This has generated a treatment protocol that now requires testing in a randomised trial. Definitive conclusions regarding efficacy need to be corroborated in future work, although the treatment appears to be safe. Assuming any treatment effect is real, these data raise the question of whether the reduction in LUTS associated with antimicrobial therapy is clinically significant.

Our PGI-I data point strongly to the overall reduction in LUTS, conferring a meaningful improvement for patients. Furthermore, relief of urinary urgency exceeded the minimal clinically important difference (MCID) described for the urgency subscale of our LUTS questionnaire [24]. Whilst no MCID has been defined for the lower urinary tract pain subscale of the instrument, the mean number of pain symptoms fell from 3.2 to 0.4, which is likely to be clinically significant. Whether the reduction in voiding symptoms amongst patients was of clinical importance is difficult to determine. The reduction in 24-h urinary frequency associated with treatment was significantly greater than that conferred by antimuscarinic medication in patients with OAB [5]. Twenty-four-hour urinary incontinence episodes did not change significantly, but baseline incontinence frequency was too low to expect a detectable change associated with treatment.

Outcome data were not generated within a study, and no formal control group is available for comparison. Nonetheless, SUI symptoms amongst patients exposed to treatment targeting other LUTS did not change. Given our understanding of the pathophysiology of SUI, we would not expect antibiotic treatment to influence these symptoms. These data provide a positive control group. In addition, our centre has collected urinary biomarker data from control individuals in prospective research studies with 12-month follow-up [25, 26]. Thirty-six women (mean age = 45.5 years; SD = 11.4) were included as controls in these studies, providing monthly MSU samples for analysis. Median pyuria expression during follow-up was zero. Statistical comparison of these control data with patient data presented here demonstrates higher levels of pyuria expression amongst patients than controls across all time points (*F* = 39; *p* = 0.0001).

Demographic variables and disease chronicity did not predict outcome in association with antibiotic therapy, and many of the baseline symptom scores did not demonstrate any predictive properties. Greater urinary urgency and frequency were predictors of less favourable outcomes, although parameter estimates suggest that their utility in clinical practice would be questionable. Higher pain scores at baseline did predict a greater reduction in overall symptoms associated with treatment. If this is a genuine finding, it may hint that pain, a central feature of the inflammatory response, is a marker of infective, treatable pathology in these patients*.*

We tried to minimise treatment duration, but symptom relapse associated with a return of pyuria often necessitated further treatment cycles. As our treatment strategy evolved, we focused on first-generation, narrow-spectrum antibiotics at maximum dose, combined with methenamine hippurate, guided by changes in symptoms and urine microscopy. Methenamine has a general bactericidal effect in the urinary tract, suppressing bacterial growth without selecting for resistant microbes. These choices are likely to generate less bacterial resistance than using conventional antibiotics alone. Our resistance data would seem to support this approach.

Symptom control usually required the maximum tolerated dose of an antibiotic and, in some patients, more than one drug. This reality is not surprising given what we now know about host/pathogen interactions in rUTI. Polymicrobial infections are not unusual [5]. Given that genomic and other more modern approaches began to reveal bacterial diversity in this patient population [2], antibiotics targeting fastidious organisms were also introduced. In addition, several common uropathogens, including *Enterococcus faecalis* [27], *Escherichia coli, Staphylococcus saprophyticus, Klebsiella pneumoniae* and *Salmonella enterica*, are known to invade urothelial cells and form intracellular bacterial communities [28]. Such reservoirs may be resistant to antibiotics present in the lumen, as many such drugs are not cell-permeant. This means that any sequestered bacteria are free to emerge later to reinitiate infection. The deeper layers of the bladder mucosa may harbour bacterial reservoirs, and cell turnover is slow. Uropathogens can also form biofilms that elaborate a polymeric capsule, conferring intrinsic antibiotic resistance. Most bacteria within these biofilms divide little, thereby failing to express a therapeutic target for most antimicrobial drugs [29]. These insights might account for the protracted treatment periods required to achieve disease regression. Recurrent relapse, experienced by some patients in association with antimicrobial withdrawal, might be explained by similar mechanisms. The failure of some patients to tolerate antimicrobial withdrawal represents a significant clinical challenge that needs to be addressed in future work.

It is worth noting that intracellular reservoirs and biofilms clinging to shed urothelial cells are unlikely to be recovered during routine MSU culture. This test samples very small volumes of urine supernatant (typically 1–10 µl), whereas infected cells settle quickly to the bottom of sample tubes. Our laboratory and others [1, 11] have found that enhanced collection methods involving collecting sediment via centrifugation provide the necessary sensitivity.

The damped oscillations in pyuria demonstrated by most patients under treatment in this study suggest that antibiotic inhibition in the lower urinary tract is partial. Damped oscillations are seen in systems in which the retarding force, in this case antimicrobial therapy, is smaller than the force it opposes [30]. The damped oscillator and long treatment periods needed support the use of maximum antibiotic doses. These oscillations also argue against our results being explained by simple regression to the mean.

In summary, the evolved treatment strategy is as follows: UTI diagnosis [7] rests on symptoms, signs and microscopic pyuria. Without the latter, we do not initiate antibiotic treatment. We combine methenamine hippurate with a first-generation, narrow-spectrum antibiotic against urinary infection to find a tolerated regime that mediates a symptomatic response and a reduction in pyuria. If urethral pain and dysuria are prominent, macrolides and tetracyclines are favoured. We continue treatment until symptom control is optimal and pyuria has cleared before trialling treatment withdrawal. More than one cycle is frequently required to achieve lasting symptom resolution.

We provide all patients who have completed their treatment with a short course of first-generation antibiotic used for urinary infection that can be self-initiated at the very first hint of symptom resurgence. Patients are advised to take 3–7 days of antimicrobial treatment, dependening on how quickly the new symptoms settle, and this approach is advocated to prevent chronic symptoms reasserting themselves after an acute infection. In the medium term, this approach seems to be effective, although we have yet to collect data on the long-term success of this strategy.

Given these data, an RCT is the next logical step. We believe that the correct design should be a comparative trial of the management protocol evolved here against treatment stipulated by current guidelines. We hope that these data will help in the design of future studies.
